# Case report: Comprehensive exploration of a novel PFKM mutation in glycogen storage disease Type VII

**DOI:** 10.3389/fgene.2024.1422908

**Published:** 2024-08-02

**Authors:** Ying Chen, Xinyu Wang, Na Ji, Qi Fang, Xin Chang, Meirong Liu

**Affiliations:** ^1^ Department of Neurology, The First Affiliated Hospital of Soochow University, Suzhou, China; ^2^ Department of Rheumatology, First Affiliated Hospital of Soochow University, Suzhou, China

**Keywords:** glycogen storage disease Type VII, PFKM, hemolysis, case report, compound heterozygous mutations

## Abstract

Glycogen Storage Disease Type VII (GSD VII) is a rare glycogen metabolism disorder resulting from mutations in the PFKM gene, inherited in an autosomal recessive manner. It is characterized by exercise intolerance, muscle cramps, myoglobinuria, compensatory hemolysis, and later onset *de novo* myasthenia and mild myopathy, contributing to its clinical heterogeneity and diagnostic challenges. Here, we report a rare case of a 17-year-old Chinese woman exhibiting substantial muscle weakness and compensated hemolysis. Muscle biopsies showed glycogen deposition, and blood tests showed hyperuricemia and significantly elevated creatine kinase. Whole genome sequencing (WGS) and whole exome sequencing (WES) identified two compound heterozygous mutations in the PFKM (NM_000289.6) gene: c.626G>A and c.1376G>A in exons 7 and 15, respectively. According to the clinical presentation, diagnostic examination, and WES results, the patient was finally diagnosed with GSDVII. The discovery of these two new PFKM mutations expands the genetic spectrum, and understanding the clinical manifestations of these mutations is critical to preventing diagnostic delays and timely intervention and treatment.

## Introduction

Glycogen Storage Disease Type VII (GSD VII) stands out as an exceedingly rare autosomal recessive glycogen storage disorder resulting from homozygous or compound heterozygous mutations in the PFKM gene, responsible for encoding the muscle phosphofructokinase (PFK) enzyme (Uyeda, 1979; Musumeci et al., 2012). PFK, the pivotal regulator of glycolysis, plays a crucial role by catalyzing the committed step, converting fructose-6-phosphate to fructose-1,6-bisphosphate (Webb et al., 2015). The human PFK is comprised of three isoenzyme subunits (muscle [M], liver [L], and platelet [P]), each encoded by distinct genes (Vora et al., 1983b).

In GSD VII, the deficiency of the muscle isoenzyme (PFK-M) manifests as exercise intolerance and compensated hemolytic anemia, with symptom severity linked to individual enzyme activity levels (Vora et al., 1983a). This disorder exhibits four distinctive clinical forms: a severe infantile form with rapidly progressing myopathy and childhood fatality; the classic childhood form marked by muscle weakness, exercise intolerance, myoglobinemia, and myoglobinuria due to varying degrees of rhabdomyolysis; a delayed-onset form observed in individuals aged 40–50, characterized by mild proximal weakness; and an exceptionally rare hemolytic form without muscle involvement, identified in a limited number of individuals (Raben and Sherman, 1995; Nakajima et al., 2002).

This study presents a detailed exploration of the clinical, biochemical, and molecular genetic features of GSD VII observed in a 17-year-old Chinese girl. Highlighted clinical manifestations, including muscle weakness and rhabdomyolysis, as well as the discovery of novel mutations in the PFKM gene, which contribute to a more comprehensive insight into the manifestations of GSD VII.

## Case description

We present the case of a 17-year-old female patient admitted to the hospital due to recurrent bilateral lower extremity soreness and weakness persisting for 3 years, with aggravation noted in the past week. The patient’s medical history revealed the onset of generalized weakness without apparent cause 3 years ago, particularly noticeable after physical activity, especially in the lower limbs. She experienced diminished motor ability, limited participation in sports, and could climb stairs up to three floors with bilateral calf pain and nausea. In September 2021, the patient sought consultation at a local gastroenterology department, where blood tests indicated elevated creatine kinase (2735 U/L), total bilirubin (82 umol/L), AST (64 U/L), and LDH (276 U/L). Despite the diagnosis of hepatic insufficiency and rhabdomyolysis, no specific treatment was initiated at the external hospital. One week prior to admission to our hospital, her condition worsened, marked by increased weakness in both lower extremities, accompanied by soreness. She could only tolerate climbing two flights of stairs.

Upon admission, the patient’s parents, who are first-degree cousins, reported no family history of hereditary conditions or similar diseases. Physical examination revealed yellow scleral discoloration, bilateral gastrocnemius pressure pain, and graded muscle strength of 5/5 in both upper limbs and 4/5 in both lower limbs. The patient demonstrated the ability to maintain upper limb planks for 1 minute and perform squats for ten repetitions.

Laboratory tests revealed a significant elevation of creatine kinase, hyperuricemia, and mild hemolysis, with specific parameters detailed in Table 1. Notably, an MRI scan of both thighs exhibited no abnormalities. This comprehensive clinical presentation underscores the complexity of the patient’s symptoms, necessitating a thorough diagnostic evaluation.

**TABLE 1:** Laboratory findings of the patient on hospitalizations. TP, Total Protein; AST, Aspartate aminotransferase; LDH, Lactate Dehydrogenase; HBDH, hydroxybutyrate dehydrogenase; HDL, High-Density Lipoprotein; APTT, activated partial thromboplastin time; FIB, focused ion beam; AT III, Antithrombin III.

	Normal values	Hospitalization
Creatine Kinase (U/L)	40–280 U/L	2005
Uric acid (umol/L)	155–357	404.5
TP (g/L)	68–88	66.1
Direct bilirubin (umol/L)	≤16.2	16.4
Indirect bilirubin (umol/L)	0–6.8	56.6
Total bilirubin (umol/L)	≤23.0	73
AST	10–31	34
LDH (U/L)	100–230	288
HBDH (U/L)	72–182	261
HDL (mmol/L)	≥1.0	0.81
Plasma APTT (sec)	25.0–31.3	23.9
Plasma FIB (g/L)	1.8–3.5	1.55
Plasma Activity changes of AT III (%)	103.2–113.8	88
CD3^+^CD8^+^ (%)	18.1–29.6	32.13
ACA-IgM (MPLU/mL)	≤12	19.408
GP210		Positive (+)

**FIGURE 1:**
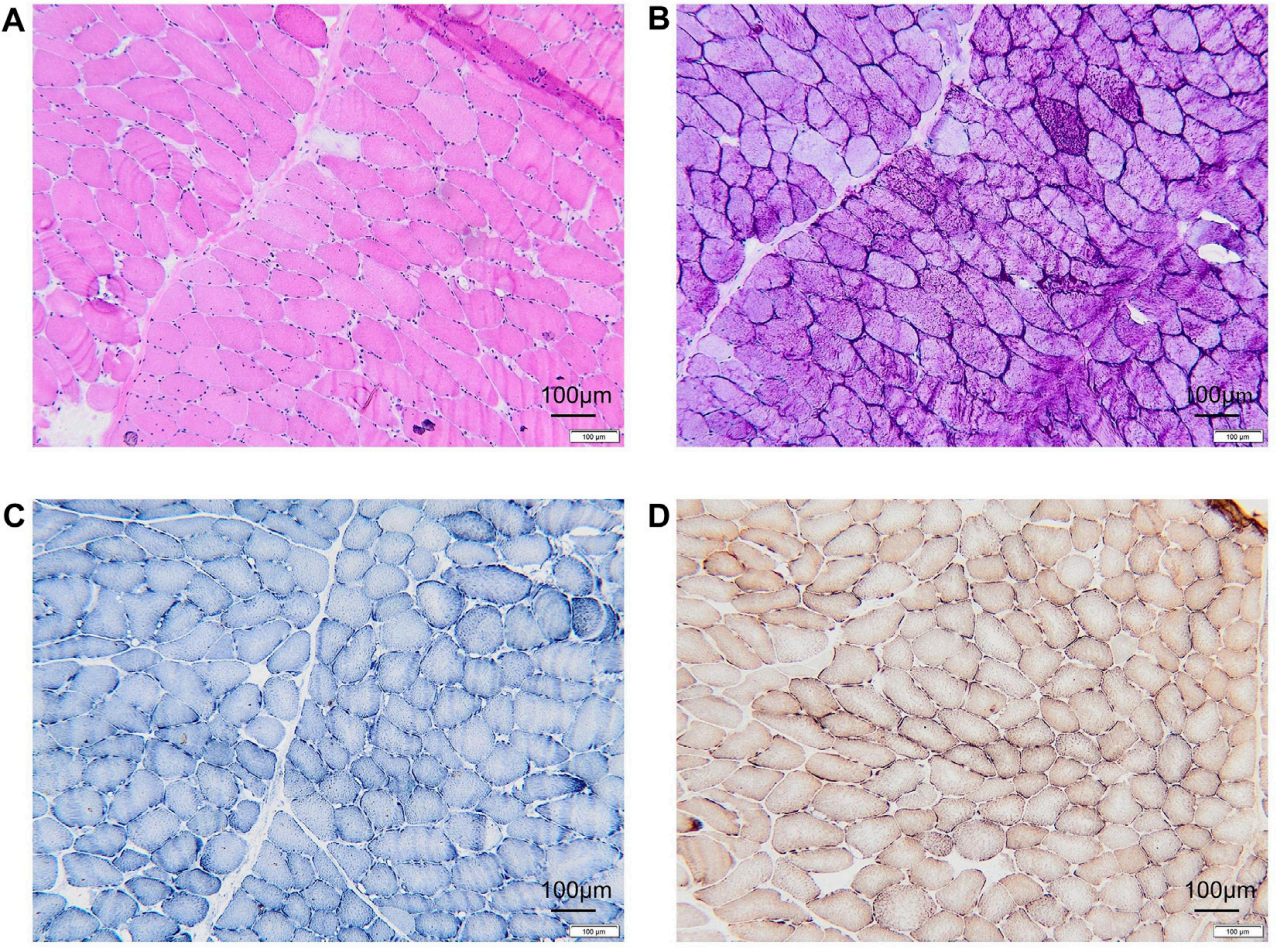
Histopathologic manifestations of the patient muscle. **(A)** Hematoxylin-eosin (HE) staining revealing vacuole formation beneath the myoplasmic membrane. **(B)** Glycogen deposition in muscle fibers, identified as PAS-positive in glycogen staining. **(C)** NADH staining indicating an absence of evident positive areas. **(D)** No abnormalities observed in COX + SDH double staining. PAS, Schiff periodic acid shiff; NADH, Nicotinamide adenine dinucleotide; COX, cytochrome c oxidase; SDH, Succinate dehydrogenase.

A muscle biopsy was performed on the medial head and fascia of the patient’s left gastrocnemius muscle. Hematoxylin and eosin (HE) staining revealed marked morphological abnormalities, including muscle fibers of variable size, predominantly small polygons and rounded shapes, with vacuole formation observed in the subplasma membrane of the muscle (Figure 1A). Periodic Acid-Schiff (PAS) staining uncovered pools of glycogen-like material of varying sizes and locations within a subset of muscle fibers. Additionally, some of these fibers, along with others lacking evident glycogen stores, exhibited signs of atrophy (Figure 1B). Nicotinamide adenine dinucleotide (NADH) staining showed no obvious abnormalities (Figure 1C). Succinate dehydrogenase (SDH) staining displayed a proliferation of mitochondria beneath the sarcolemma (Figure 1D). Skeletal muscle biopsy showed the presence of myogenic injury and the presence of vacuoles and glycogen staining under the muscle plasma membrane suggests a possible glycogen metabolism-related myopathy, but other myogenic injuries such as myositis and mitochondrial myopathy need to be ruled out.

Subsequently, a serum immunoassay revealed a high percentage of 32.13% CD3^+^CD8^+^, and autoimmune liver disease antibodies were detected. The autoimmune myositis antibody profile test demonstrated positivity for GP210, and the anti-cardiolipin antibody assay (ACA) reported an ACA-IgM level of 19.408 MPLU/mL. These findings provide a comprehensive understanding of the patient’s immunological and autoimmune profile, serving as a crucial component in the diagnostic evaluation. The patient was admitted to the hospital and given ursodeoxycholic acid for hepatoprotective treatment, and oral dose is 0.25 g/capsule BID.

**FIGURE 2:**
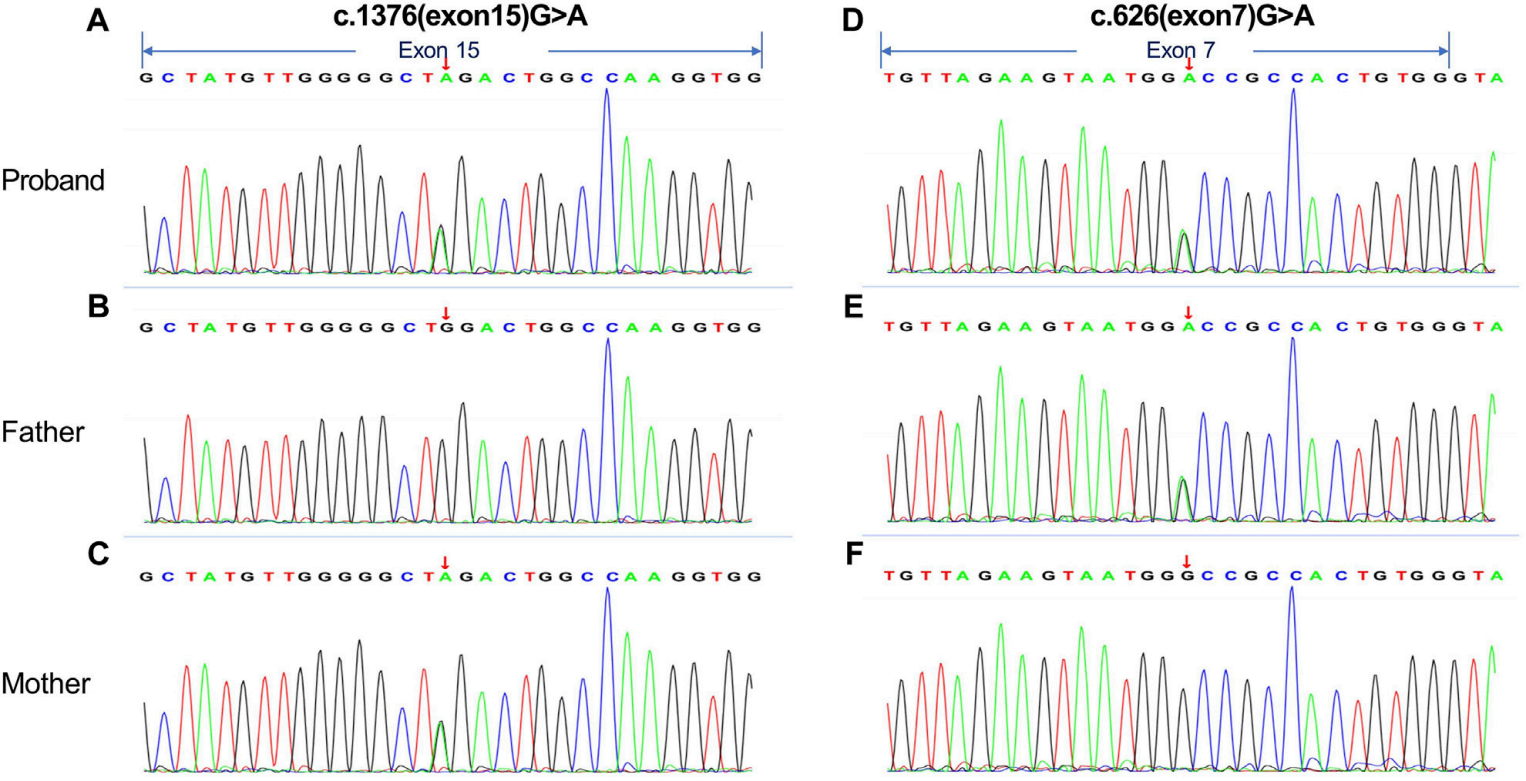
Whole genome sequencing (WGS) and whole exome sequencing (WES) were performed on the proband and her parents. **(A, D)** The results indicated that the proband had compound heterozygous mutations in the PFKM gene: **(C)**1376 (exon15) G>A variant in exon 15 and **(C)**626 (exon7) G>A variant in exon 7. **(B, C and E, F)** Sanger traces for PCR of her parents. The **(C)**1376 (exon15) G>A on exon15 and **(C)**626 (exon7) G>A on exon 7 come from the mother and father, respectively. The arrows in the figure indicate exonic regions.

For further diagnosis, whole genome sequencing (WGS) and whole exome sequencing (WES) were performed to identify the genetic lesions responsible for the disease phenotype. The majority of WGS + WES was conducted by the Beijing Zhiying Eastern Translational Medicine Research Center. The main steps are as follows: firstly, DNA is extracted and sequencing libraries are constructed by random interruption method. The constructed sequencing libraries are up-sequenced with no less than 99% genome coverage. Finally, the data were analyzed using bioinformatics and clinical information analysis techniques. Approximately 99.72% of the sequencing reads mapped to the human genome hg19, revealing two compound heterozygous disease-associated variants of PFKM (NM_000289.6) in the preclears: the maternally inherited variant c.1376 (exon15) G>A and the paternally inherited variant c.626 (exon7) G>A. The c.1376G>A variant in exon 15 resulted in a mutation of amino acid Trp to Ter (p. Trp562Ter, 322) at position 562 of the protein encoded by the PFKM gene, while the c.626 (exon7) G>A variant in exon 7 resulted in Gly to Asp (p. Gly312Asp) (Figures 2A,D). We also performed Sanger sequencing of her parents. The 1376 (exon15) G>A on exon15 and 626 (exon7) G>A on exon 7 come from the mother and father, respectively. (Figures 2B–C,E–F).

The variants c.1376 (exon15) G>A and c.626 (exon7) G>A were not reported in the 1,000 Genomes database, Genome Aggregation Database (gnomAD), and the HGMD professional database. Additionally, these mutations were not identified in 100 healthy Chinese control individuals. Following the American College of Medical Genetics and Genomics (ACMG) guidelines (2019), evidence for pathogenic or potentially pathogenic variants is categorized into four levels: very strong (PVS1), strong (PS1-4), moderate (PM1-6), and supportive (PP1-5). In this case, the missense mutation in c.1376 (exon15) G>A, leading to the amino acid Trp variant becoming Ter at protein position 562 encoded by the PFKM gene, is classified as pathogenic (PVS1+PM2+PM3) (Richards et al., 2015). When considering the patient’s clinical characteristics, the deleterious nature of the PFKM gene variant in this case aligns with the observed phenotype.

## Discussion

In this study, we identified two novel compound heterozygous mutations in the PFKM gene, c.1376 (exon15) G>A and c.626 (exon7) G>A, in a patient diagnosed with GSD VII (also known as PFKM deficiency), by utilizing WGS and WES. GSD VII is a rare autosomal recessive genetic metabolic disorder characterized by a high degree of clinical heterogeneity in the form of exercise intolerance, muscle cramps, compensated hemolysis, hyperuricemia, and potentially myoglobinuria (Sherman et al., 1994; Nakajima et al., 1995; Raben et al., 1995).

We successfully revealed two previously unreported mutations by applying WES and WGS, thus broadening our understanding of the genetic status of GSD VII-related genes. The PFKM gene is located at chromosome 12q13 and includes 23 exons spanning approximately 30 kb (Yang et al., 2022). To date, 27 mutations in the PFKM gene have been reported in the human genome database, including deletions, duplications, intronic deletions, insertions, and single nucleotide changes (Nakajima et al., 1990; Richards et al., 2015). Vives-Corrons JL et al. first described a case of GSDVII with a novel mutation in PFKM: c.926A>G; p. Asp309Gly, which the authors hypothesized would severely affect enzyme catalysis and thus explain the observed enzyme deficiency (Vives-Corrons et al., 2013). Auranen et al. reported on two patients with GSD, both of whom had adolescent-onset impaired motor performance with spasticity and rare myoglobinuria. Muscle biopsies showed glycogen accumulation, but GSD was ruled out due to phosphofructokinase immunohistochemistry. However, WES testing confirmed the diagnosis of GSDVII by showing the pathogenic, pure PFKM gene defect, R39Q, in both siblings (Auranen et al., 2015). There are also historical cases, such as those reported by Layzer et al. (1969) and Tsujino et al. (1994), which have played a key role in elucidating the complexity of muscular PFK deficiency and associated erythrocyte hemolysis (Layzer et al., 1969; Tsujino et al., 1994). It is shown that mutations in the PFKM gene can suggest disease onset in GSD VII as well as help us to diagnose it in time.

Presently, there exists no effective treatment for GSD Type VII, but the evolving field of gene therapy holds promise for future interventions. Despite the generally benign nature of GSD VII, the potential complication of rhabdomyolysis can pose life-threatening risks. The clinical presentation of our patient aligns with previously reported cases, reinforcing the need for consideration of hereditary metabolic disorders in individuals exhibiting such symptoms (Agamanolis et al., 1980). Comprehensive diagnostic measures, including blood biochemical tests, skeletal muscle biopsy, and enzymatic tests, are instrumental in achieving an accurate diagnosis. Subsequent genetic testing serves as a crucial confirmation step, as highlighted in our study (Danon et al., 1981; Servidei et al., 1986; Auranen et al., 2015). Presently, there exists no effective treatment for GSD Type VII, but the evolving field of gene therapy holds promise for future interventions (Kishnani et al., 2019).

## Conclusion

In conclusion, our study contributes novel insights into the genetic basis of GSD VII by identifying two previously unreported mutations in the PFKM gene. The patient improved with treatment, with no generalized muscle tenderness and grade 5 muscle strength in the extremities. These findings advance our understanding of the disorder and may have implications for genetic diagnosis, counseling, and potential therapeutic avenues in the future. The comprehensive approach to diagnosis and management outlined in our study reinforces the importance of considering hereditary metabolic disorders in clinical practice.

## Data Availability

The original contributions presented in the study are included in the article/Supplementary Material, further inquiries can be directed to the corresponding authors.
