# Supplementary material for: Case report: Comprehensive exploration of a novel PFKM mutation in glycogen storage disease Type VII
Source: Front Genet. 2024 Aug 2;15:1422908. doi: 10.3389/fgene.2024.1422908 (PMC11327043; doi:10.3389/fgene.2024.1422908)
Supplement: Supplementary file 1 [file DataSheet1.PDF]

CARE case report guidelines

CARE Checklist of information to include when writing a case report

(cc) BY-NC-ND

Topic	Item	Checklist item description	Reported on Line
Title	1	The diagnosis or intervention of primary focus followed by the words "case report"	Line 2-3
Key Words	2	2 to 5 key words that identify diagnoses or interventions in this case report, including "case report"	Line 28-29
Abstract (no references)	3a	Introduction: What is unique about this case and what does it add to the scientific literature?	Line 14-19
	3b	Main symptoms and/or important clinical findings	Line 19-21
	3c	The main diagnoses, therapeutic interventions, and outcomes	Line 24-26
	3d	Conclusion—What is the main "take-away" lesson(s) from this case?	Line 26-29
Introduction	4	One or two paragraphs summarizing why this case is unique (**may include references**)	Line 33-50
Patient Information	5a	De-identified patient specific information.	Line 58
	5b	Primary concerns and symptoms of the patient.	Line 58-65
	5c	Medical, family, and psycho-social history including relevant genetic information	Line 65-68
	5d	Relevant past interventions with outcomes	Line 68-70
Clinical Findings	6	Describe significant physical examination (PE) and important clinical findings.	Line 73-79
Timeline	7	Historical and current information from this episode of care organized as a timeline	None
Diagnostic Assessment	8a	Diagnostic testing (such as PE, laboratory testing, imaging, surveys).	Line 80-119
	8b	Diagnostic challenges (such as access to testing, financial, or cultural)	Line 120-145
	8c	Diagnosis (including other diagnoses considered)	Line154-156
	8d	Prognosis (such as staging in oncology) where applicable	None
Therapeutic Intervention	9a	Types of therapeutic intervention (such as pharmacologic, surgical, preventive, self-care)	Line 119-120
	9b	Administration of therapeutic intervention (such as dosage, strength, duration)	Line 120-121
	9c	Changes in therapeutic intervention (with rationale)	None
Follow-up and Outcomes	10a	Clinician and patient-assessed outcomes (if available)	Line 209-210
	10b	Important follow-up diagnostic and other test results	None
	10c	Intervention adherence and tolerability (How was this assessed?)	None
	10d	Adverse and unanticipated events	None
Discussion	11a	A scientific discussion of the strengths AND limitations associated with this case report	Line 155-162
	11b	Discussion of the relevant medical literature **with references**.	Line 163-205
	11c	The scientific rationale for any conclusions (including assessment of possible causes)	Line 155-159
	11d	The primary "take-away" lessons of this case report (without references) in a one paragraph conclusion	Line 207-215
Patient Perspective	12	The patient should share their perspective in one to two paragraphs on the treatment(s) they received	Line 209-210
Informed Consent	13	Did the patient give informed consent? Please provide if requested	Yes ☑ No ☐
